# Microvascular and proteomic signatures overlap in COVID-19 and bacterial sepsis: the MICROCODE study

**DOI:** 10.1007/s10456-022-09843-8

**Published:** 2022-06-20

**Authors:** Alexandros Rovas, Konrad Buscher, Irina Osiaevi, Carolin Christina Drost, Jan Sackarnd, Phil-Robin Tepasse, Manfred Fobker, Joachim Kühn, Stephan Braune, Ulrich Göbel, Gerold Thölking, Andreas Gröschel, Jan Rossaint, Hans Vink, Alexander Lukasz, Hermann Pavenstädt, Philipp Kümpers

**Affiliations:** 1 Department of Medicine D, Division of General Internal and Emergency Medicine, Nephrology, and Rheumatology, University Hospital Münster, Albert-Schweitzer Campus 1, 48149 Münster, Germany; 2 Department of Medicine A, Hematology, Oncology and Pulmonary Medicine, University Hospital Münster, Albert-Schweitzer Campus 1, 48149 Münster, Germany; 3 Department of Cardiology and Angiology, University Hospital Münster, Albert-Schweitzer Campus 1, 48149 Münster, Germany; 4 Department of Medicine B for Gastroenterology, Hepatology, Endocrinology, Clinical Infectiology, University Hospital Münster, Albert-Schweitzer Campus 1, 48149 Münster, Germany; 5 Center for Laboratory Medicine, University Hospital Münster, Albert-Schweitzer Campus 1, 48149 Münster, Germany; 6 Institute of Virology, University Hospital Münster, Von-Stauffenberg-Straße 36, 48151 Münster, Germany; 7 Departmenf of Intensive Care and Emergency Medicine, St. Franziskus-Hospital GmbH, Hohenzollernring 70, 48145 Münster, Germany; 8 Department of Anaesthesiology and Critical Care, St. Franziskus-Hospital GmbH, Hohenzollernring 70, 48145 Münster, Germany; 9 Department of Internal Medicine and Nephrology, University Hospital Münster Marienhospital Steinfurt, Mauritiusstr. 5, 48565 Steinfurt, Germany; 10 Department of Pulmonology, Clemens Hospital, Düesbergweg 124, 48153 Münster, Germany; 11 Department of Anaesthesiology, Intensive Care and Pain Medicine, University Hospital Münster, Albert-Schweitzer Campus 1, 48149 Münster, Germany; 12 Department of Physiology, Cardiovascular Research Institute Maastricht, Maastricht University, 6229 ER Maastricht, the Netherlands

**Keywords:** Microcirculation, Microvascular dysfunction, Sepsis, COVID-19, Proteomic signature, Biomarker

## Abstract

**Aims:**

Although coronavirus disease 2019 (COVID-19) and bacterial sepsis are distinct conditions, both are known to trigger endothelial dysfunction with corresponding microcirculatory impairment. The purpose of this study was to compare microvascular injury patterns and proteomic signatures in COVID-19 and bacterial sepsis patients.

**Methods and results:**

This multi-center, observational study included 22 hospitalized adult COVID-19 patients, 43 hospitalized bacterial sepsis patients, and 10 healthy controls from 4 hospitals. Microcirculation and glycocalyx dimensions were quantified via intravital sublingual microscopy. Plasma proteins were measured using targeted proteomics (Olink). Coregulation and cluster analysis of plasma proteins was performed using a training-set and confirmed in a test-set. An independent external cohort of 219 COVID-19 patients was used for validation and outcome analysis. Microcirculation and plasma proteome analysis found substantial overlap between COVID-19 and bacterial sepsis. Severity, but not disease entity explained most data variation. Unsupervised correlation analysis identified two main coregulated plasma protein signatures in both diseases that strictly counteract each other. They were associated with microvascular dysfunction and several established markers of clinical severity. The signatures were used to derive new composite biomarkers of microvascular injury that allow to predict 28-day mortality or/and intubation (area under the curve 0.90, *p* < 0.0001) in COVID-19.

**Conclusion:**

Our data imply a common biological host response of microvascular injury in both bacterial sepsis and COVID-19. A distinct plasma signature correlates with endothelial health and improved outcomes, while a counteracting response is associated with glycocalyx breakdown and high mortality. Microvascular health biomarkers are powerful predictors of clinical outcomes.

**Graphical abstract:**

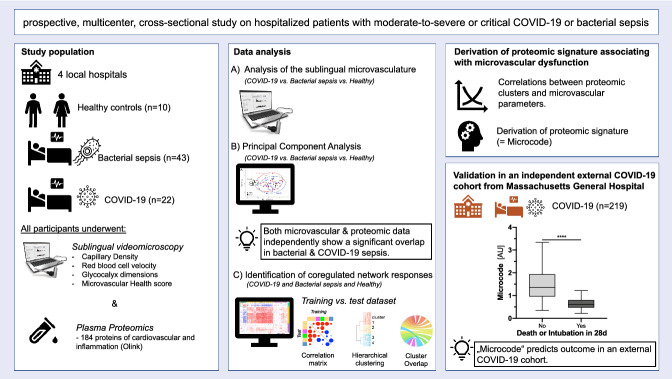

**Supplementary Information:**

The online version contains supplementary material available at 10.1007/s10456-022-09843-8.

## Introduction

Since the beginning of the coronavirus disease 2019 (COVID-19) pandemic, evidence has emerged that COVID-19 is a vascular rather than purely respiratory illness [1]. While direct viral infection of endothelial cells (ECs) with severe acute respiratory syndrome coronavirus 2 is controversial [2–4], some evidence indicates that EC injury in severely ill COVID-19 patients is secondary to the systemic inflammatory host response [1].

Numerous studies have investigated inflammatory markers in patients with severe COVID-19 and identified several biomarkers potentially associated with outcome. Among them are powerful activators of the endothelium such as interleukin (IL) 6, tumor necrosis factor (TNF-) α, vascular endothelial grow factor (VEGF-) A, and angiopoietin (Angpt-) 2. They are known to facilitate vascular hyperpermeability and microthrombosis leading to acute respiratory distress syndrome in severe COVID-19 [5–11]. Thus, the concept of a systemic inflammatory host response that drives the microthrombotic phenotype seen in COVID-19, is widely accepted.

Microvascular dysfunction is a hallmark of inflammation in bacterial sepsis [12]. Although COVID-19 and bacterial sepsis are distinct conditions, we hypothesized a common biological response that mediates microvascular damage. The aim of this proof-of-concept study was to compare microvascular injury and plasma proteins in patients with COVID-19 and bacterial sepsis to find differences and similarities between both inflammatory diseases.

## Methods

### Study design and study population

The current multi-center, prospective, observational, cross-sectional study took place in the University Hospital Münster and three local academic teaching hospitals. After written informed consent was obtained, adult hospitalized patients needing admission to the intensive care units (ICU-critical disease) and intermediate care wards (IMC-moderate/severe disease) because of COVID-19 infection or confirmed bacterial sepsis (sepsis-3 definition) [13] were prospectively enrolled in a non-consecutive manner. After initial resuscitation sublingual videomicroscopy was performed. Plasma samples were obtained, centrifuged, and stored at − 80 °C until analysis. Exclusion criteria were pregnancy or local oral mucosal inflammation. Ten healthy randomly selected age-matched volunteers served as controls, after undergoing a thorough clinical examination and laboratory blood tests. Some of the participants were already included in previous microvascular studies [9, 14, 15]. This study was approved by the relevant ethics committee (amendments of 2016–073-f-S) and was performed in accordance with the Declaration of Helsinki.

### In vivo assessment of sublingual microcirculation and glycocalyx dimensions

A sidestream dark field camera (CapiScope HVCS, KK Technology, Honiton, UK) coupled with GlycoCheck™ software (Microvascular Health Solutions Inc., Alpine, UT, USA) was used to visualize passing red blood cells (RBCs) in the sublingual microvasculature (microvessels’ diameter 4–25 µm) at the bedside as previously described in detail [15, 16]. Based on the RBC dynamics in the valid vascular segments, the software calculates the following variables, which were successfully validated in the past [14, 15, 17]:

*Perfused boundary region* (PBR, in µm) expresses the dynamic lateral movement of RBCs into the permeable part of the endothelial glycocalyx layer, an inverse parameter of endothelial glycocalyx dimensions. The higher the PBR values, the more diminished the glycocalyx dimensions.

*Capillary density* (in 10^−2^ mm/mm^2^) was defined as the vascular density of vessels with a diameter ≤ the diameter of a single RBC (diameter ~ 7–8 µm [18]; capillary density diameter ≤ 7 µm).

*RBC velocity* (in µm/sec) can be determined in individual vessel segments in an automatic manner via cross correlation of longitudinal RBC intensity profiles between frames of recorded videos.

*The combination of microcirculation and glycocalyx variables* enables the estimation of microvascular health score MVHS_*static*_; higher values indicate healthier microvasculature.

### Targeted plasma proteomics, circulating glycocalyx markers and angiopoietin-2

The Olink (Uppsala, Sweden) “inflammation1” and “cardiovascular2” proteomic panels comprise 92 proteins each. Seven proteins are common to both panels. A total of 184 proteins in 75 samples (COVID-19, bacterial sepsis, healthy) were measured in one batch to avoid technical variation. Briefly, the Olink proximity extension assay uses two specific oligonucleotide-labeled antibodies per protein (“probes”). When the two probes are in proximity a new PCR target sequence is formed via a proximity-dependent DNA polymerization event. The resulting sequence is subsequently detected and quantified using standard real-time quantitative PCR, as previously reported [19]. Measurements were conducted in triplicate. Results are reported as arbitrary units on a log2 scale. The proteins included in each panel, measurement details, and validation data are available online (www.olink.com/downloads).

Plasma levels of the glycocalyx-core protein syndecan-1 (Diaclone, Besançon, France) and the endothelial-specific proinflammatory mediator Angpt-2 (R&D Systems, Minneapolis, USA) were measured using commercially available enzyme-linked immunosorbent assay kits in accordance with the manufacturer’s instructions.

### External validation: Massachusetts General Hospital COVID-19 cohort

The proteomic signature derived from the study cohort was validated in a public database of adult COVID-19 patients admitted to Massachusetts General Hospital (MGH, Boston, Massachusetts, USA, https://www.olink.com/mgh-covid-study) [20]. The inclusion criteria were clinical concern of COVID-19 upon emergency department admission, and acute respiratory distress with at least one of the following: tachypnea ≥ 22 breaths/minute; oxygen saturation ≤ 92% on room air; requirement for supplemental oxygen; positive-pressure ventilation. The primary study outcome was a composite endpoint of 28-day mortality and/or intubation, and a total of 219 blood samples drawn on day 3 were assessed in conjunction with the primary study outcome.

### Statistical analysis

Data are presented as absolute numbers, percentages, and medians with corresponding 25th and 75th percentiles (interquartile ranges; IQRs), as appropriate. The non-parametric Mann–Whitney-*U* and Kruskal–Wallis test with Dunn’s multiple comparisons, and the chi-square test were used to compare groups, as appropriate. To correct for multiple testing in comparisons of proteome data between bacterial sepsis and COVID-19, the false discovery rate approach of Benjamini–Hochberg was used, with a q-value of 1% deemed significant. Pearson’s correlation coefficient (*r*) was used to assess correlations between variables. Receiver-operating characteristic analysis was used to estimate areas under the curve (AUCs). All tests used were two-sided, and statistical significance was set at *p* < 0.05. SPSS (IBM Corporation, Armonk, NY, USA, v.26) and GraphPad Prism (GraphPad Prism Software Inc., San Diego, CA, USA, v.8.4.3) were used for statistical analyses.

#### Correlation analysis, principal component analysis and clustering

Pearson was used to correlate one protein with all other proteins using the proteomics dataset across all participants. The protein data were not normally distributed (Shapiro–Wilk test), thus Pearson’s correlation with bootstrapping (iteratively removing one participant) was applied (see Fig. 2 for details). There were no missing values in the dataset. The weakest correlation coefficient between two proteins after bootstrapping was displayed in a similarity matrix, whereby the circle size indicates the p value and the color indicates the correlation coefficient (red = positive, blue = negative). The matrix (rows and columns) was clustered by euclidean distance with average linkage.

The principal components in the multivariate dataset were calculated using singular value decomposition with imputation (pre-normalized data, no transformation), and visualized using ClustVis [21].

To determine the optimal number of clusters in each data set, the elbow method was applied using k means. It uses the within-cluster sum of square as a function of the number of clusters and creates a plot whereby the elbow of the curve is considered the optimal threshold. This analysis yielded an optimal cluster number of 3 in the training, test and validation set (Supplemental Fig. 1).

#### Term enrichment, and functional annotation

All significant clusters were subjected to functional annotation and term enrichment analysis using Metascape [22], which allows functional enrichment analysis (GO/KEGG/Reactome terms, canonical pathways, hallmark gene sets) and overlap analysis across multiple gene/protein lists. It uses the hypergeometric test and Benjamini–Hochberg *p* value correction to identify the terms that contain a greater number of genes of an input list than expected by chance. A subset of representative terms was selected and converted into a network layout. Each term is represented by a node, whose size represents the number of input proteins of that term. Terms with a similarity score > 0.3 are linked by an edge, and edge thickness is proportional to the similarity score. The network was visualized with Cytoscape (v.3.1.2) [23].

## Results

Our dataset consisted of 43 patients with bacterial sepsis and 22 with COVID-19 (Table 1, Supplemental Table 1). There were no significant differences between median [IQR] age (68 [57–79] vs. 63 [52–76] years, *p* = 0.12), sex (*p* = 0.14), or disease severity (Sequential Organ Failure Assessment [SOFA] score 9 [4–12] vs. 6 [2–12], *p* = 0.22) in the two groups. Ten healthy individuals served as controls.

**Table 1:** Baseline characteristics

Variables	Healthy controls	Bacterial sepsis	COVID-19	*p* value*
Number of participants (*n*)	10	43	22	–
Female sex (*n*; %)	7 (70)	14 (32.6)	3 (13.6)	0.14
Age (years, median (IQR))	51 (27–69)	68 (57–79)	63 (53–76)	0.12
BMI (kg/m^2^, median (IQR))	23 (21.5–25.8)	25.3 (21.1–27.7)	26.5 (23.4–30.1)	0.15
Charlson Comorbidity Index (points, median (IQR))	–	2 (1–3)	1 (0–3)	0.14
ICU (*n*; %)	–	33 (76.7)	15 (68.2)	0.55
SOFA score (points, median (IQR))	–	9 (4–12)	6 (2–12)	0.22
Mechanical ventilation (*n*; %)	–	19 (44.2)	13 (59.1)	0.30
Inhospital mortality (*n*; %)	–	13 (30.2)	6 (27.3)	0.99
MAP (mmHg)	92.3 (89.2–99.4)	73.7 (66.7–87.3)	78.2 (71.9–90.2)	0.29
Laboratory data (median (IQR))				
CRP (mg/dl)	0.5	21.6 (12.8–31.8)	12.2 (4.5–21.9)	0.02
Ferritin (µg/l)	106 (18–255)	589 (156–977)	962 (454–1451)	0.03
PCT (ng/ml)	0.05	7.3 (0.7–46.7)	0.6 (0.1–3.2)	< 0.0001
Creatinine (mg/dl)	0.85 (0.68–0.95)	1.9 (1.2–3.1)	0.8 (0.7–1.5)	0.003

*BMI* body mass index, *CRP* C-reactive protein, *IQR* interquartile range, *MAP* mean arterial pressure, *PCT* procalcitonin, *SOFA score* sequential organ failure assessment score

*p value was calculated between bacterial sepsis and COVID-19 cohort

### Microvascular phenotyping by quantitative sublingual video microscopy

**Fig. 1:**
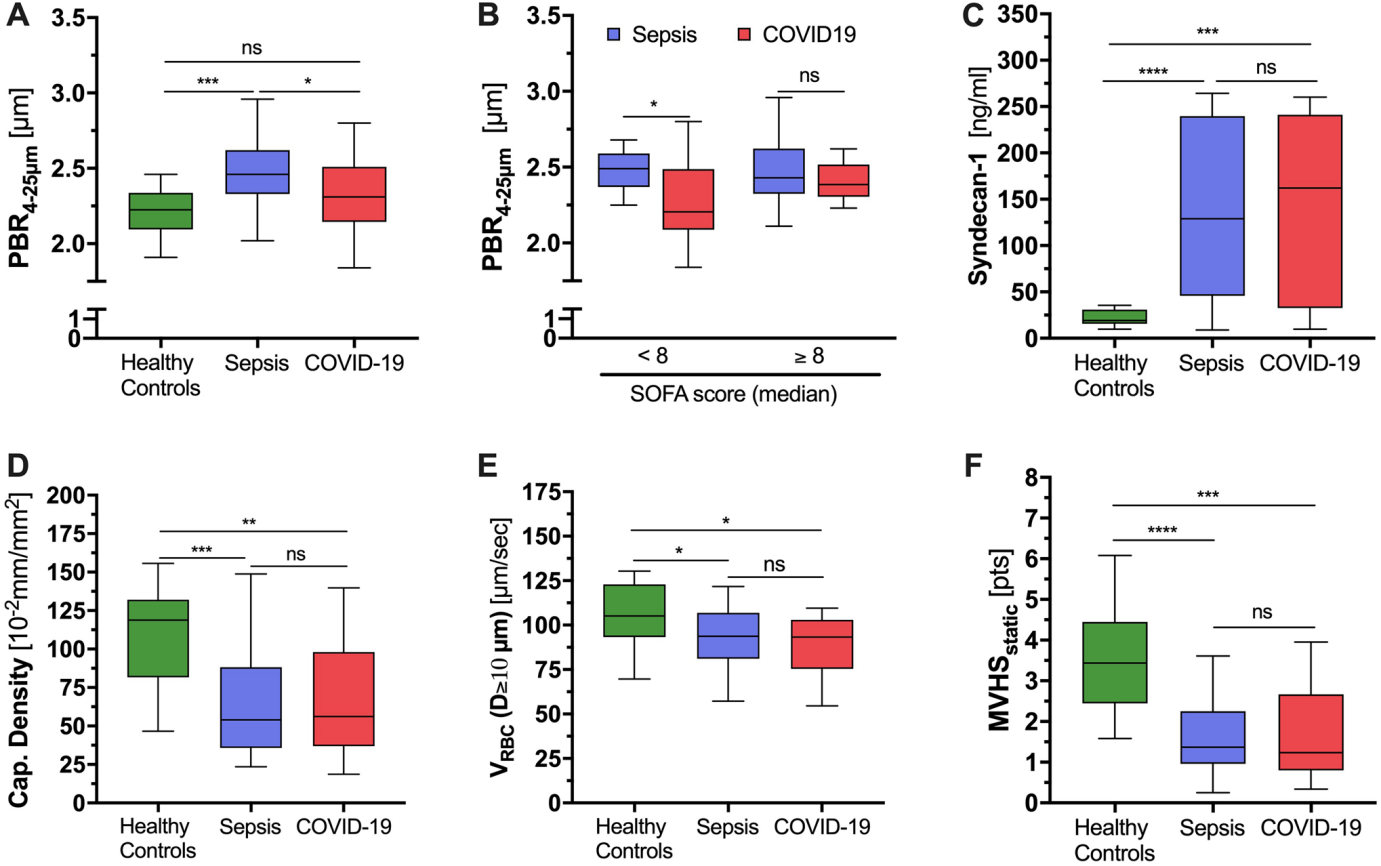
Microvascular phenotyping by quantitative sublingual videomicroscopy and in vitro glycocalyx markers in patients with bacterial sepsis (blue) or COVID-19 (red). Boxplots of perfused boundary region (PBR) based on **A** disease entity and **B** disease severity. **C** Boxplots of syndecan-1, a circulating glycocalyx marker. Boxplots of **D** capillary density (4–7 µm), **E** red blood cell velocity in the feed vessels, and **F** microvascular health score (MVHS). Ten apparently healthy subjects (green) were used as controls. *ns* not significant. *p < 0.05, **p < 0.01, ***p < 0.001, ****p < 0.0001

The perfused boundary region (PBR) is a surrogate marker for glycocalyx breakdown as measured by sublingual video microscopy. Bacterial sepsis patients exhibited significantly higher PBR_4–25 µm_ values (*i.e.*, thinner endothelial glycocalyx) than COVID-19 patients and healthy controls (Fig. 1A). However, severely ill COVID-19 and bacterial sepsis patients (dichotomized by the overall median SOFA score of ≥ 8) showed a similar PBR_4–25 µm_ increase, indicating that the severity of inflammation associates with glycocalyx injury in both conditions (Fig. 1B). This finding was further corroborated by equally elevated plasma levels of shed syndecan-1 in both patient groups (Fig. 1C).

Both bacterial sepsis and COVID-19 patients exhibited > 50% reductions in capillary density, and significantly lower RBC velocity in feed vessels compared to healthy controls (Fig. 1D, E). The median [IQR] MVHS_static_ was similarly decreased in both diseases compared to controls (bacterial sepsis 1.37 [0.96–2.25] vs. COVID-19 1.24, [0.80–2.67], *p* = 0.61) (Fig. 1F). Together, the sublingual microscopy data suggest similar patterns and severity of microvascular dysfunction in bacterial sepsis and COVID-19.

### Unsupervised correlation analysis of plasma proteomics

**Fig. 2:**
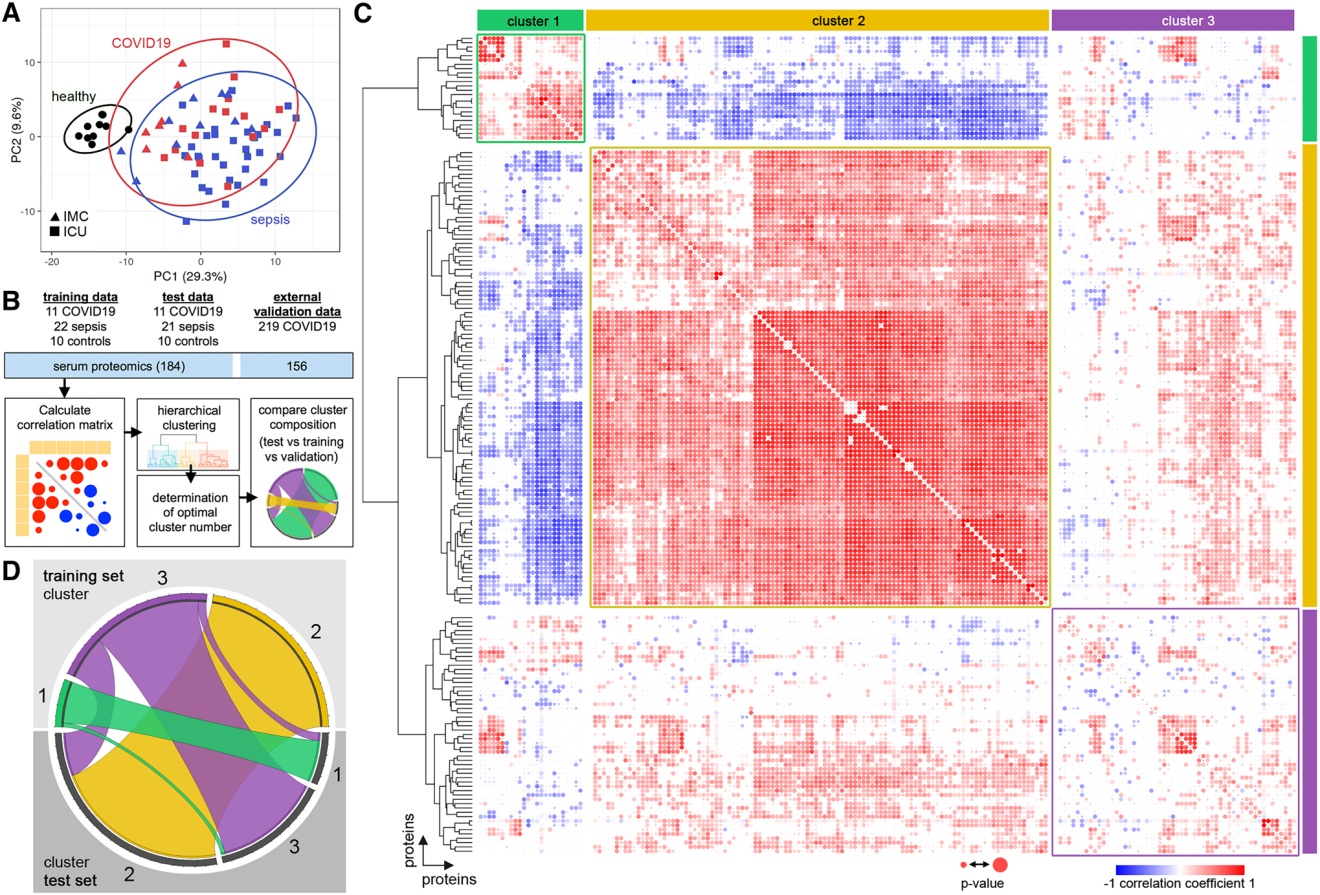
Unsupervised systems analysis to identify coregulated network responses. **A** Principal component (PC) analysis. The ellipses show a probability of 95% that a new datapoint from the same group is located inside the ellipse. **B** Overview of the workflow. Twenty-two COVID-19 and forty-three bacterial sepsis patients were divided into a matched training-set and a matched test-set. Ten apparently healthy individuals were used as controls. **C** Pearson correlation coefficients of all parameters were calculated with a bootstrapping algorithm. Briefly, it iteratively calculates the Pearson’s correlation coefficient for each data matrix minus one sample. For *n* = *x* samples, *x* similarity matrices will be calculated each time excluding one sample. The correlation coefficient closest to “0” (the weakest correlation) in x correlations for one pair of parameters will be used as a result and the confidence level can be determined. To test the significance of each pairwise correlation a Student’s *t*-distribution was calculated with a significance threshold of 0.05. The final result of the 184 proteins in the training-set was plotted as a similarity matrix of all serum proteins with the color indicating the correlation coefficient (red = high positive correlation, blue = high negative correlation) and the dot size indicating the significance. Significance was calculated using the two-sided *t*-test and is expressed as square size. **D** Cluster overlap between the training-set and the test-set. The three main clusters identified in the training-set remained significant in both the training-set and the test-set (coregulated protein clusters in the test-set and the external validation cohort are shown in Supplemental Figs. 3 and 4, respectively)

We performed targeted proteomics of 184 inflammation- and metabolism-related proteins using blood plasma. The principal component (PC) analysis revealed substantial overlap between bacterial sepsis and COVID-19 patients, but a clear distinction between healthy controls and patients (Fig. 2A, Supplemental Fig. 2). In line with this finding, after correcting for multiple tests only 7 proteins changed significantly in COVID-19 patients compared to bacterial sepsis (Table 2).

**Table 2:** Proteins showing a difference between bacterial sepsis and COVID-19

Protein	*q* value	COVID-19^a^
FGF21	0.001085	↓
GDF2	0.001085	↑
IL24	0.001339	↓
SORT1	0.003627	↑
IL6	0.004503	↓
CCL23	0.004503	↓
TGM2	0.008966	↑

^a^Compared to bacterial sepsis

As we found vast similarities between COVID-19 and sepsis using microvascular and proteomic data, we hypothesized a similar biological host response. To elucidate co-regulated protein signatures, we applied unsupervised correlation and clustering analysis (workflow overview—Fig. 2B). To generate a test and training dataset, all patients were matched by SOFA score, resulting into two groups (Fig. 2B). There were no significant differences in clinical or laboratory data (Supplemental Table 2).

After plotting correlation coefficients as a heat map (184 × 184 proteins) and sorting by euclidean distance with average linkage, small and large coregulated protein clusters emerged along the diagonal that reflected mostly positive (red) and few negative (blue) correlations (Fig. 2C). Three main clusters (hereafter named cluster 1 to 3) emerged using the training-set. The same analysis was performed independently with the test-set, which also resulted in three protein clusters with largely similar composition compared to the training-set (Fig. 2C, D). For external validation, an independently published Olink protemics dataset was used containing only COVID-19 patients (MGH cohort, see methods). Coregulated protein clusters showing significant overlap with clusters 1 and 2 of the test and training-set were identified (Supplemental Fig. 4). These data suggest a common host response in inflammatory disease with serum proteins forming coregulated signatures.

### Functional annotation and term enrichment analysis

**Fig. 3:**
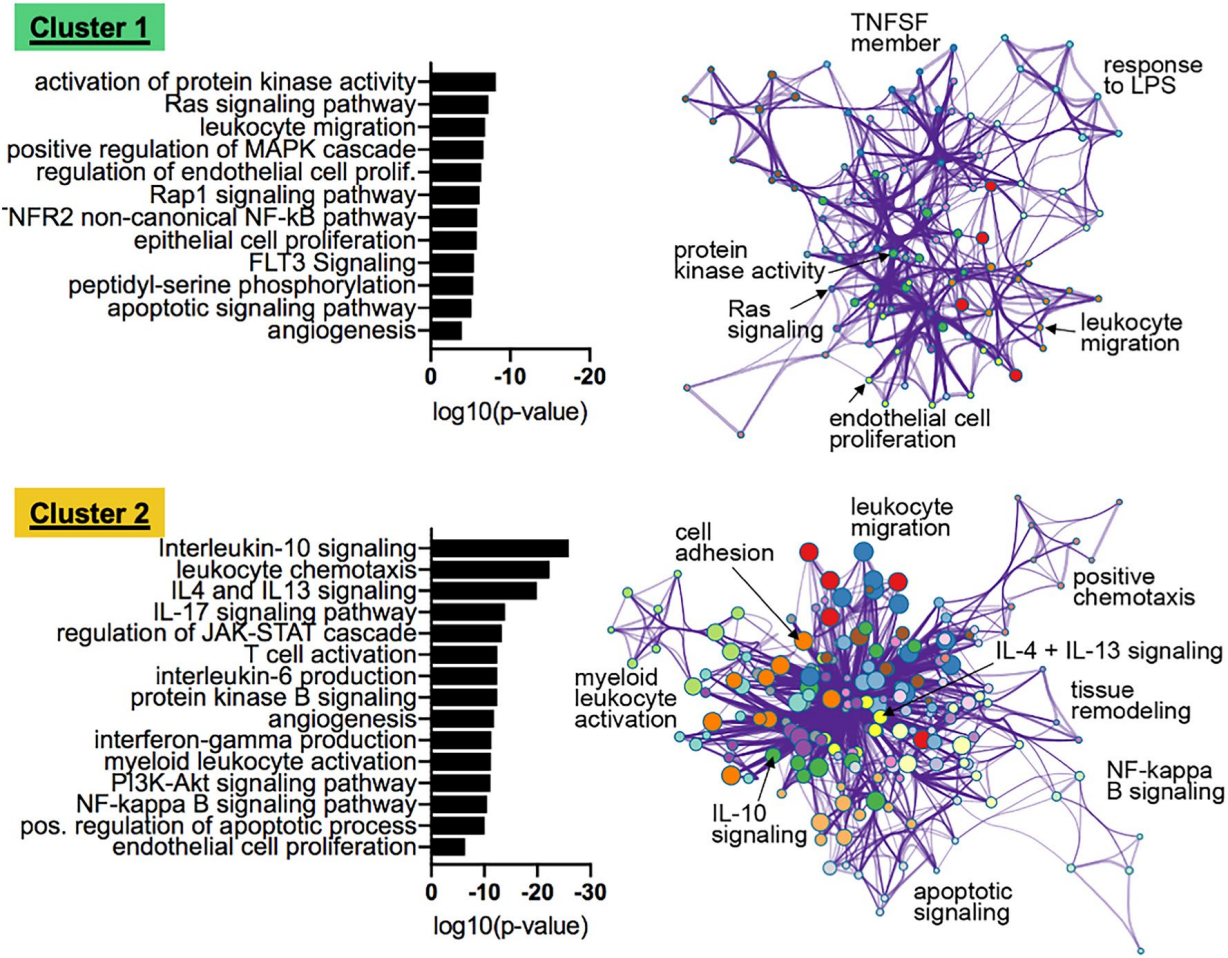
Significant pathways engaged in clusters 1 and 2 according to functional annotation and term enrichment analysis. Proteins from clusters 1 and 2 were subjected to functional annotation and term enrichment analysis using Metascape. The network was visualized with Cytoscape (v.3.1.2)

Cluster 1 contained a total of 23 unique proteins, including von Willebrand factor-cleaving protease (ADAMTS13), Angpt-1, and VEGF-D. Cluster 2 contained 100 unique proteins, including IL2, IL6, IL8, IL10, IL14, IL16 and various inflammatory mediators (Supplemental Table 3). Functional annotation and term enrichment analysis of the proteins in each cluster suggested specific biological functions and revealed the engagement of different pathways (Fig. 3). IL10/IL13/IL4 signaling and interferon gamma production were detected in cluster 2, whereas Ras, Ras-related protein 1, and mitogen-activated protein kinase signaling were enriched in cluster 1, among others. Cluster 3 did not show any clinical correlations (see below) and was excluded from the functional analysis.

**Table 3:** Correlations of cluster 1 (mean) and cluster 2 (mean) with clinical and laboratory variables

Variable	Mean arterial pressure	NE dosis	Ferritin	CRP	Creatinine	Syndecan-1	Angpt-2
Cluster 1—Mean	0.39**	− 0.34**	− 0.52**	− 0.76**	− 0.34**	− 0.43**	− 0.73**
Cluster 2—Mean	− 0.54**	0.39**	0.27**	0.53**	0.61**	0.66**	0.71**
Cluster 3—Mean	− 0.16	0.05	0.12	− 0.20	− 0.12	0.21	0.01

Pearson correlation was used

*Angpt-2* angiopoietin-2, *BMI* body mass index, *CRP* C-reactive protein, *NE* norepinephrine, *SOFA score* sequential organ failure assessment score

*p < 0.05, **p  < 0.01

### Associations between proteomic clusters and disease severity, disease entity, and microvascular parameters

As all proteins in each protein cluster are positively correlated, we calculated the mean cluster concentration of all cluster proteins for each patient. Interestingly, the normalized means of clusters 1 and 2 were negatively correlated with each other (*r* = − 0.52, *p* < 0.0001; Supplemental Fig. 5), suggesting opposite or intertwined biological responses. The means of cluster 3 did not correlate with microvascular, laboratory or clinical parameters (Supplemental Fig. 6, Table 3) and therefore was excluded from further analysis. Regardless of the SOFA score, there were no significant associations between clusters 1 and 2 and the disease entity (bacterial sepsis/COVID-19) (Supplemental Fig. 7A, C).

Cluster 1 and 2 correlated significantly with several established markers of critical/acute illness in sepsis and COVID-19 patients, including mean arterial pressure, C-reactive protein, ferritin, creatinine, norepinephrine dose and SOFA score (Table 3). For example, cluster 1 was negatively correlated with the SOFA score (*r* = − 0.66, *p* < 0.0001), whereas cluster 2 was positively correlated (*r* = 0.74, *p* < 0.0001) (Supplemental Fig. 7B, D). Similar findings were made regarding the number of dysfunctional organ systems (Supplemental Fig. 8) and microvascular measurements. Both protein clusters were moderately correlated with capillary density and PBR_4–25 µm_ (Supplemental Fig. 6). In line with these data, both clusters were strongly correlated with the glycocalyx breakdown-associated proteins syndecan-1 and Angpt-2 (Table 3). Again, cluster 1 was positively correlated with microvascular health and negatively correlated with microvascular injury; and cluster 2 vice versa. These findings suggest that glycocalyx integrity is linked to the systemic host response during inflammatory disease.

### Derivation of a composite biomarker of microvascular dysfunction

**Fig. 4:**
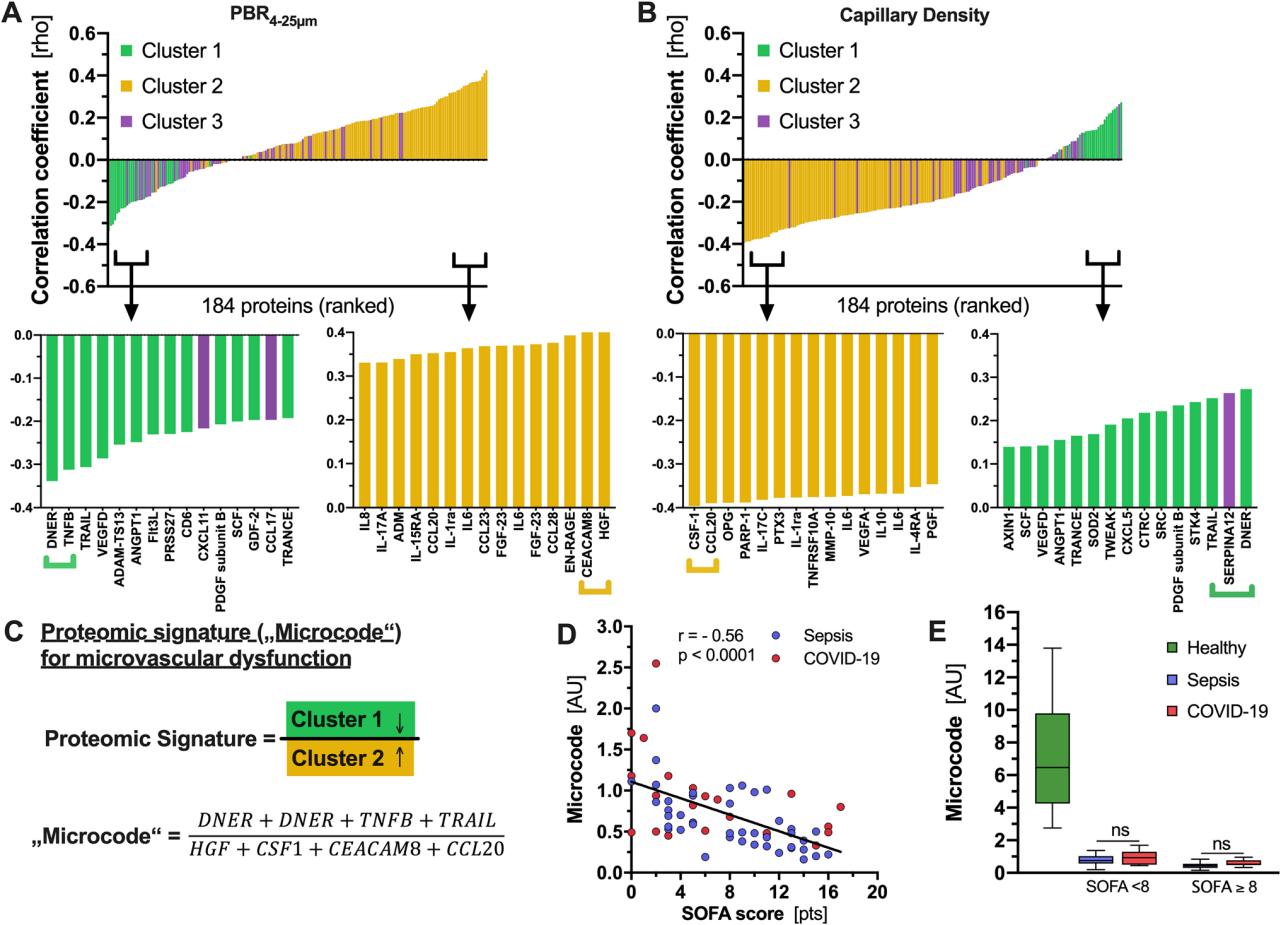
Derivation of a proteomic signature (“*Microcode*”) of microvascular dysfunction. Correlations between the 184 proteins in the proteomics analysis and **A** PBR 4–25 μm and **B** capillary density in a ranked manner. Cluster 1 (green) correlates with intact microcirculation (low PBR, high capillary density) and cluster 2 (yellow) correlates with damaged microcirculation (high PBR, low capillary density). **C** Combination of the “top eight” proteins (four per cluster) derive a proteomic signature (“*Microcode*”) of microvascular dysfunction. The proteins of each cluster were normalized and their quotient was calculated per subject using the formula: ${{{\text{Cluster}}\,1\,{\text{proteins}}} \mathord{\left/ {\vphantom {{{\text{Cluster}}\,1\,{\text{proteins}}} {{\text{Cluster}}\,2\,{\text{proteins}}}}} \right. \kern-\nulldelimiterspace} {{\text{Cluster}}\,2\,{\text{proteins}}}}$.. **D** Correlation of “*Microcode*”values with SOFA score in bacterial sepsis and COVID-19 patients. **E** Boxplots showing median [IQR] “*Microcode*” values and disease severity and entity. *ns* not significant. *p < 0.05, **p < 0.01, ***p < 0.001, ****p < 0.0001

A second approach was to identify individual proteins that correlate with microvascular measurements. Therefore, we plotted the Pearson correlation coefficients of PBR_4–25 µm_ (Fig. 4A) and capillary density (Fig. 4B) with each of the 184 proteins in ascending order. After coloring the cluster membership of each protein (as shown in Fig. 2), most cluster 1 proteins (green) correlated with low PBR and high capillary density, indicating a healthy microcirculation. In contrast, most cluster 2 proteins (yellow) correlated with high PBR and low capillary density, indicating microvascular dysfunction. This approach highlights that microvascular injury is mirrored by specific changes in the serum proteome.

We next aimed to develop a composite biomarker based on plasma proteins that correspond to microvascular health. Given that cluster 1 and cluster 2 were inversely correlated, the following quotient served as a proteomic signature of microvascular dysfunction (hereafter referred to as “*Microcode*”) (Fig. 4C):

$${\text{Top}}{{\,{\text{Cluster}}\,1\,{\text{proteins}}} \mathord{\left/ {\vphantom {{\,{\text{Cluster}}\,1\,{\text{proteins}}} {{\text{Top}}\,{\text{Cluster}}\,2\,{\text{proteins}}}}} \right. \kern-\nulldelimiterspace} {{\text{Top}}\,{\text{Cluster}}\,2\,{\text{proteins}}}} .$$

Because cluster 1 decreased with disease severity and cluster 2 increased, the *Microcode* values decreased with increasing microvascular dysfunction. *Microcode* values were not different between COVID-19 and bacterial sepsis, but associated with disease severity in both diseases (Fig. 4D-E). As our study was not designed for outcome analysis, we used the external MGH cohort.

### “Microcode” predicts outcome in the validation set

**Fig. 5:**
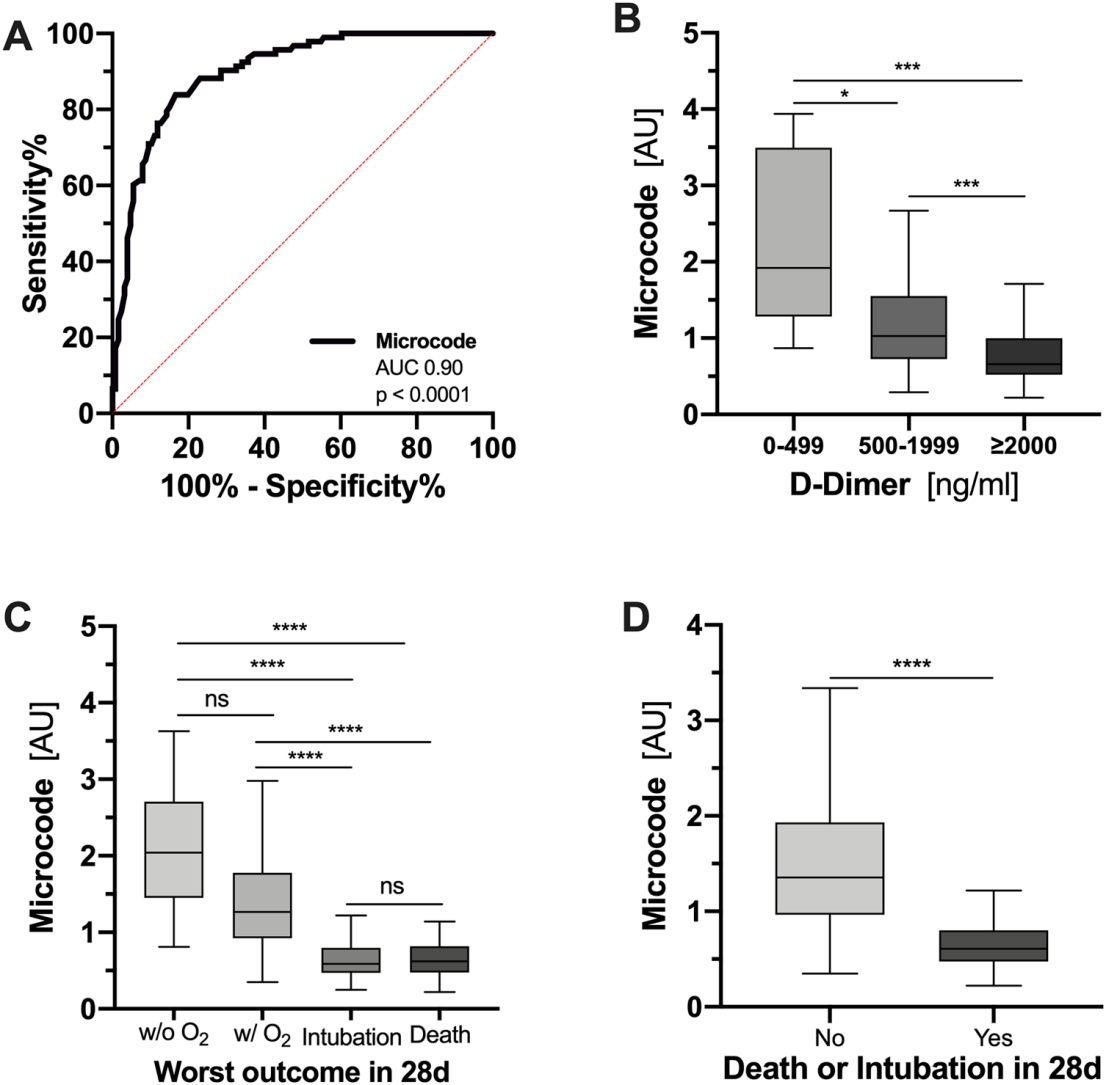
External validation of “*Microcode*” signature in an independent COVID-19 cohort from Massachusetts General Hospital (*n* = 219). **A** Receiver-operating characteristic curves showing the predictive capacity of the *Microcode*. **B**–**D** Boxplots of *Microcode* values classified based on **B** correspondent D-dimer values, **C** the worst outcome in the 28 days of hospitalization, and **D** the composite endpoint of 28-day mortality and/or intubation. *AU* arbitrary units, *ns* not significant. *p < 0.05, **p < 0.01, ***p < 0.001, ****p < 0.0001

*Microcode* was then applied to the external COVID-19 validation cohort (MGH), which annotates proteome and outcome data. Regarding the composite endpoint 28-day mortality and/or intubation, *Microcode* performed similar to the known COVID-19 biomarker IL6 [24] (AUC [95% CI] 0.90 [0.86–0.94, *p* < 0.0001] vs. IL6 0.88 [0.83–0.92, *p* < 0.0001]) (Fig. 5A). Low *Microcode* values associated with higher D-dimer levels, a complicated clinical course, and a poorer outcome (Fig. 5B–D). Of note, proteomic signatures for PBR and capillary density associated independently with disease severity (Supplemental Fig. 9).

## Discussion

The current analysis demonstrates substantial overlap of both, microvascular and proteomic phenotypes in critically ill patients with COVID-19 and bacterial sepsis. To date very few studies have investigated differences and similarities between COVID-19 and bacterial sepsis. Regarding the inflammatory and immune response, the results are inconsistent, presumably because the two entities are inherently difficult to match [25–28]. In the present study, we observed differences in the routine laboratory data; bacterial sepsis patients showed higher procalcitonin (PCT) and C-reactive protein (CRP) values, while COVID-19 patients showed higher ferritin values. These differences have been reported previously and might be attributed to the alternative pathophysiological models of organ dysfunction between the two entities. Briefly, compared to bacterial sepsis, SARS-CoV-2 probably causes a direct viral injury, associates with lymphopenia and is responsible for a virus-induced immunosuppression [29]. In line with past studies, IL-6 levels were significantly higher in our sepsis cohort compared to COVID-19 [30, 31]. However, IL-6 receptor blockers (e.g., tocilizumab) suppressing the so-called cytokine storm are still recommended in COVID-19 patients with systemic inflammation [32], but not in bacterial sepsis.

Despite the above observed differences, our data clearly show that microvascular and proteome signatures of both diseases were very similar. In both entities glycocalyx and microcirculatory impairment were associated with clinical severity and specific proteome signatures. Both COVID-19 and bacterial sepsis patients exhibited virtually the same changes when compared to healthy controls. These concordant deviations from the healthy state were identified via unsupervised systems analysis and clustered into two groups, each of which correlated with disease severity. The correlation between a proinflammatory protein response (cluster 2) and microvascular injury was not surprising, but another signature (cluster 1) was also identified that corresponded with microvascular health. Our data imply a system’s biology concept with co-regulated host responses across different inflammatory disease. This also allowed to develop composite biomarkers that predicted mortality in an external, independent cohort of COVID-19 patients. To our knowledge, this is the first clinical biomarker that was derived from microvascular health parameters.

Our novel approach of the microcirculation enabled us to compare the microvascular phenotype of COVID-19 patients with that of bacterial sepsis patients, and to correlate proteomic data with PBR and capillary density. Although individual protein correlations were only moderate, a prototypical composite biomarker consisting of several proteins with the strongest associations was able to predict outcome in an independent, external COVID-19 cohort. This constitutes proof-of-concept that the devised *Microcode* signature can be used for risk stratification in COVID-19 patients. As there is no corresponding public proteome library derived from bacterial sepsis patients, it is not possible to test the microvascular signature in a dedicated bacterial sepsis cohort.

Notably the protein signature identified is purely associative, and no causal relationships can be inferred. Most proteins in the prototypical signature have been attributed to the regulation of different components of the immune system, and only hepatocyte growth factor and TNF-β have been studied in relation to vascular barriers and endothelial function, respectively [33–36]. Among the other candidates significantly correlated with glycocalyx thickness or capillary density there were several proteins with strong vascular connections, such as VEGF, Angpt-1, and ADAMTS13. These candidates, as well as other pathways identified via functional annotation and term enrichment analysis, can be used as a reasonable starting point for planning further mechanistic experiments.

The current study had some limitations. First, the inclusion of patients in the training and test cohorts represents a cross-sectional design. Although the wide range of patients with varying disease severity is certainly advantageous with respect to correlation analysis, this design was not suitable for the analysis of clinical outcomes. Second, the proteome data contains mainly a priori selected vascular and inflammatory proteins and does not represent the entire patients’ proteome. Third, we cannot exclude that the lack of differences between COVID-19 and bacterial sepsis might have been partially influenced by the limited sample size. However, our study is hypothesis generating and *Microcode* should be further confirmed in future trials. Fourth, it is unclear how representative and robust our *Microcode signature* is regarding outcome prediction over time, because patients were only enrolled in a cross-sectional fashion after initial resuscitation of bacterial sepsis or COVID-19-related hospitalization. Fifth, although routine microbiological sampling was performed in the COVID-19 group, we cannot exclude the possibility of bacterial superinfections in the COVID-19 group, which may have partially influenced the results. However, the prevalence of bacterial co-infections in COVID-19 is considered rather low [37, 38] and our COVID-19 cohort had a low median PCT value (0.6 ng/ml), which argues against overt co-infections.

## Conclusion and outlook

Despite the above limitations the data clearly indicate that COVID-19 and bacterial sepsis share common proteomic signatures and features of microvascular damage. Integrating multi-omic data in clinical studies is a promising approach to decipher systemic host responses and microvascular damage, and develop new diagnostic and therapeutic concepts in inflammatory disease.

## Supplementary Information

Below is the link to the electronic supplementary material.

Supplementary file1 (JPG 231 kb)

Supplementary file3 (JPG 417 kb)

Supplementary file3 (PNG 311 kb)

Supplementary file4 (PNG 761 kb)

Supplementary file5 (TIFF 297 kb)

Supplementary file6 (TIFF 857 kb)

Supplementary file7 (TIFF 586 kb)

Supplementary file8 (TIFF 439 kb)

Supplementary file9 (TIFF 586 kb)

Supplementary file10 (DOCX 28 kb)

## Data Availability

The datasets used and/or analyzed during the current study are available from the corresponding author on reasonable request. Data of the external cohort were provided by the MGH Emergency Department COVID-19 Cohort (Filbin, Goldberg, Hacohen) with Olink Proteomics and are available online.

## Abbreviations

Angpt: Angiopoietin
AUC: Area under the curve
COVID-19: Coronavirus disease 2019
CRP: C-reactive protein
EC: Endothelial cell
IL: Interleukin
IQR: Interquartile range
MVHS: Microvascular health score
PBR: Perfused boundary region
PC: Principal component
PCT: Procalcitonin
RBC: Red blood cell
SOFA: Sequential Organ Failure Assessment
TNF: Tumor necrosis factor
VEGF: Vascular endothelial growth factor
