# Supplementary figures and images for: Microvascular and proteomic signatures overlap in COVID-19 and bacterial sepsis: the MICROCODE study
Source: Angiogenesis. 2022 Jun 20;25(4):503–15. doi: 10.1007/s10456-022-09843-8 (PMC9208353; doi:10.1007/s10456-022-09843-8)

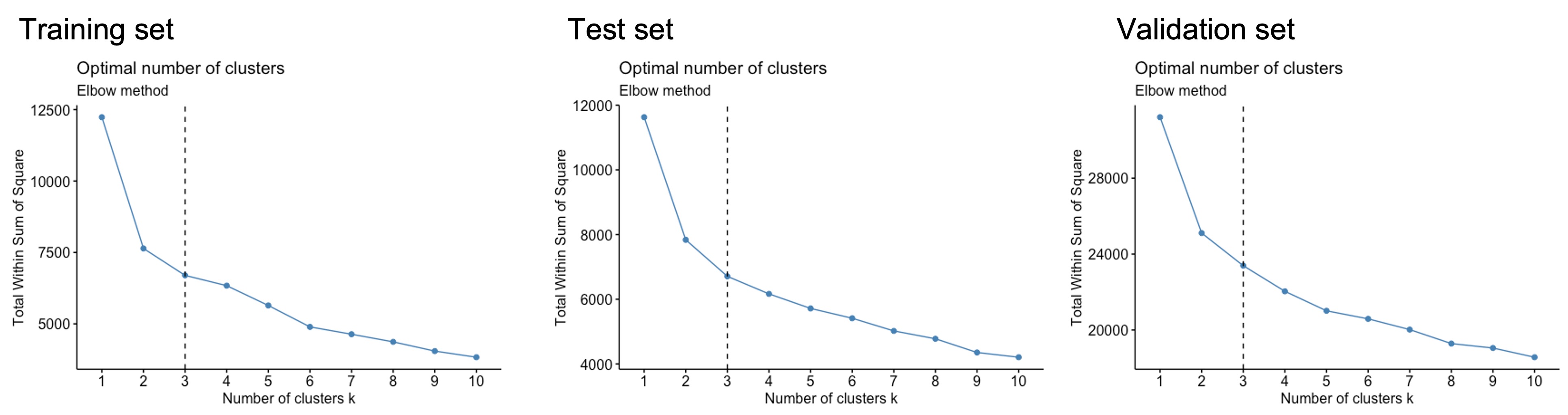

Supplement: Supplementary file 1 — Supplementary file1 (JPG 231 kb) [file 10456_2022_9843_MOESM1_ESM.jpg]

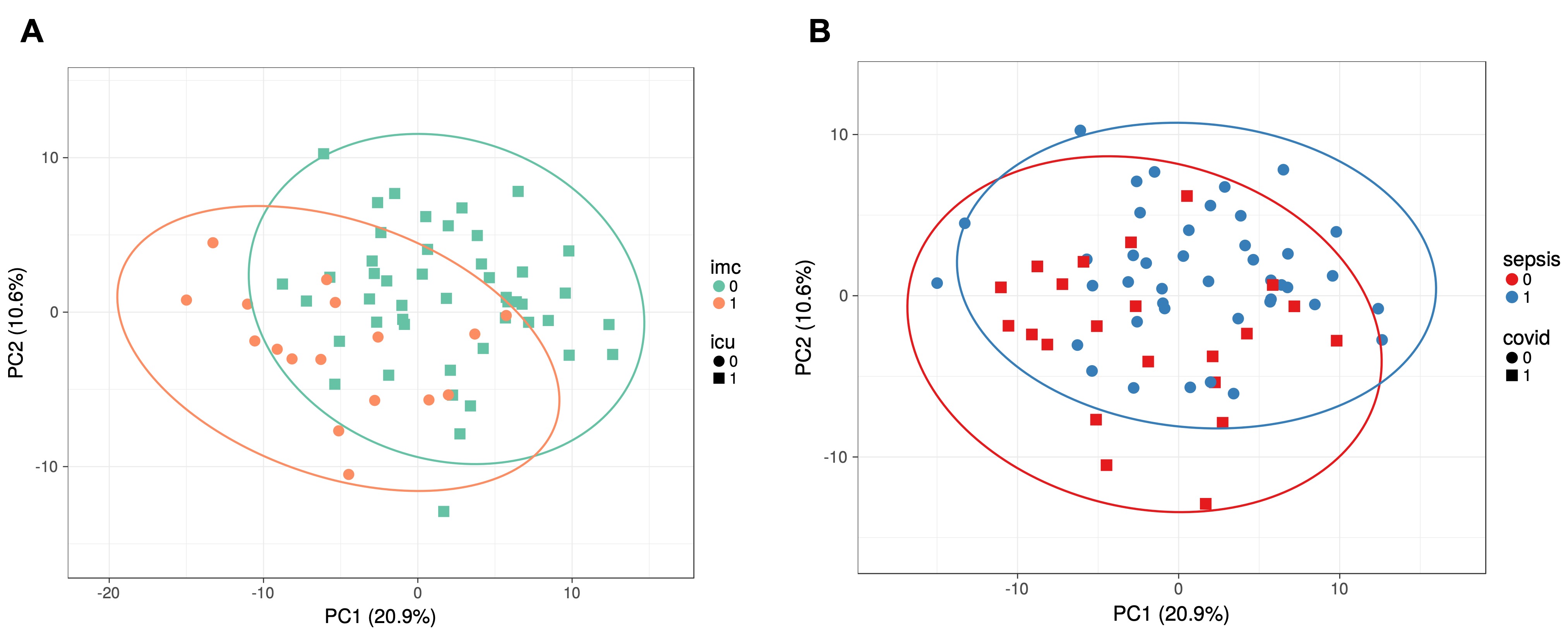

Supplement: Supplementary file 2 — Supplementary file3 (JPG 417 kb) [file 10456_2022_9843_MOESM2_ESM.jpg]

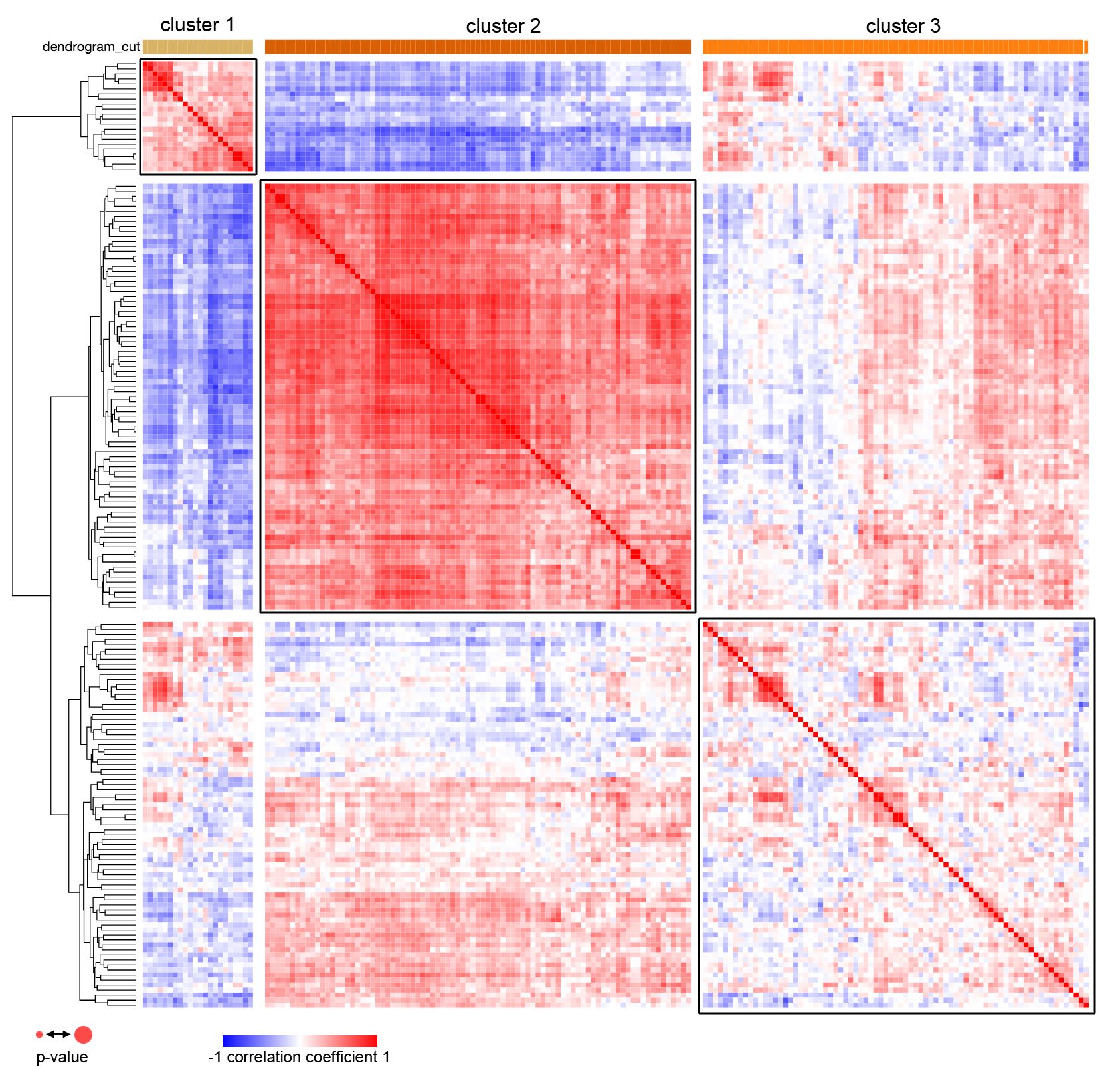

Supplement: Supplementary file 3 — Supplementary file3 (PNG 311 kb) [file 10456_2022_9843_MOESM3_ESM.png]

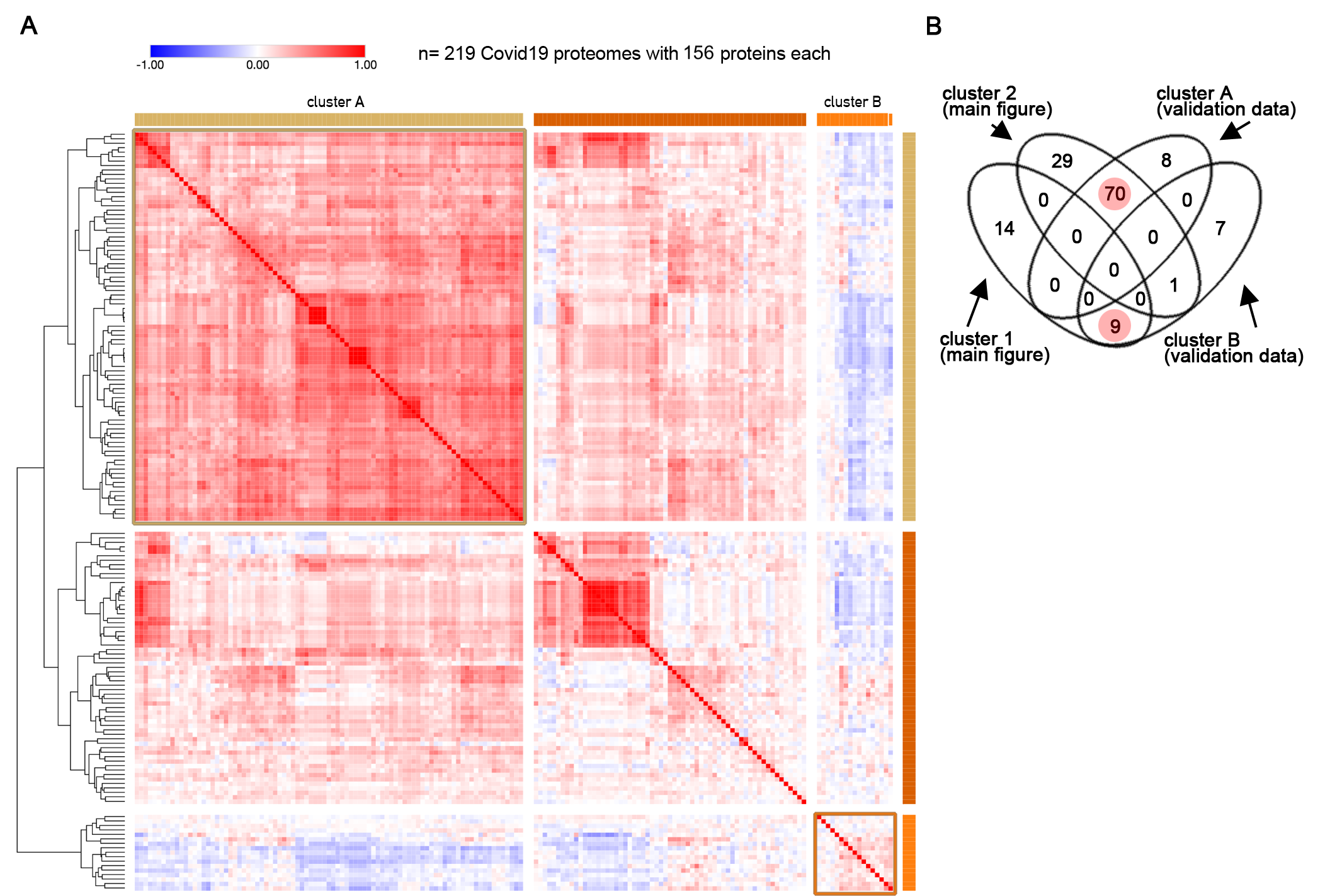

Supplement: Supplementary file 4 — Supplementary file4 (PNG 761 kb) [file 10456_2022_9843_MOESM4_ESM.png]

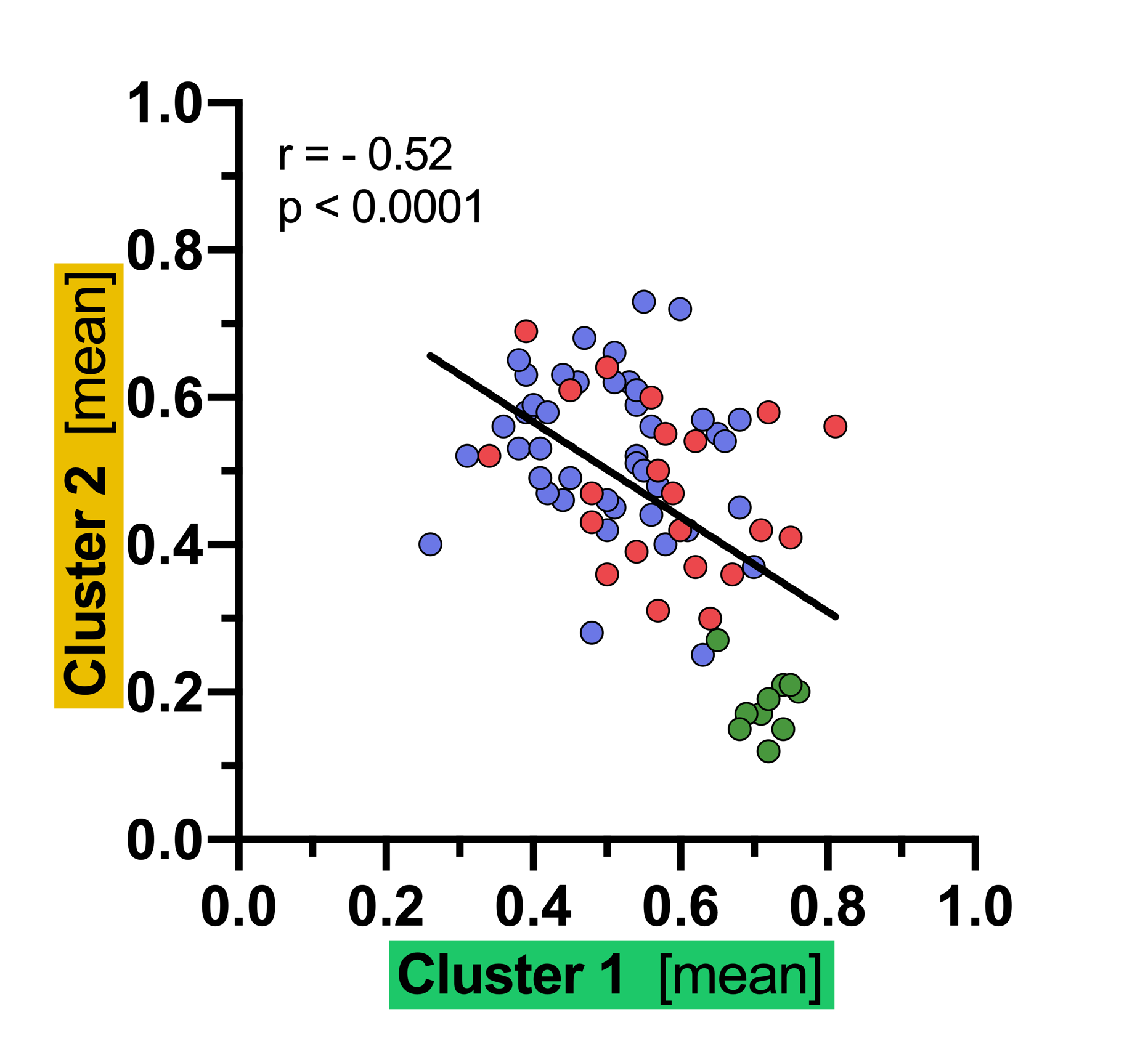

Supplement: Supplementary file 5 — Supplementary file5 (TIFF 297 kb) [file 10456_2022_9843_MOESM5_ESM.tiff]

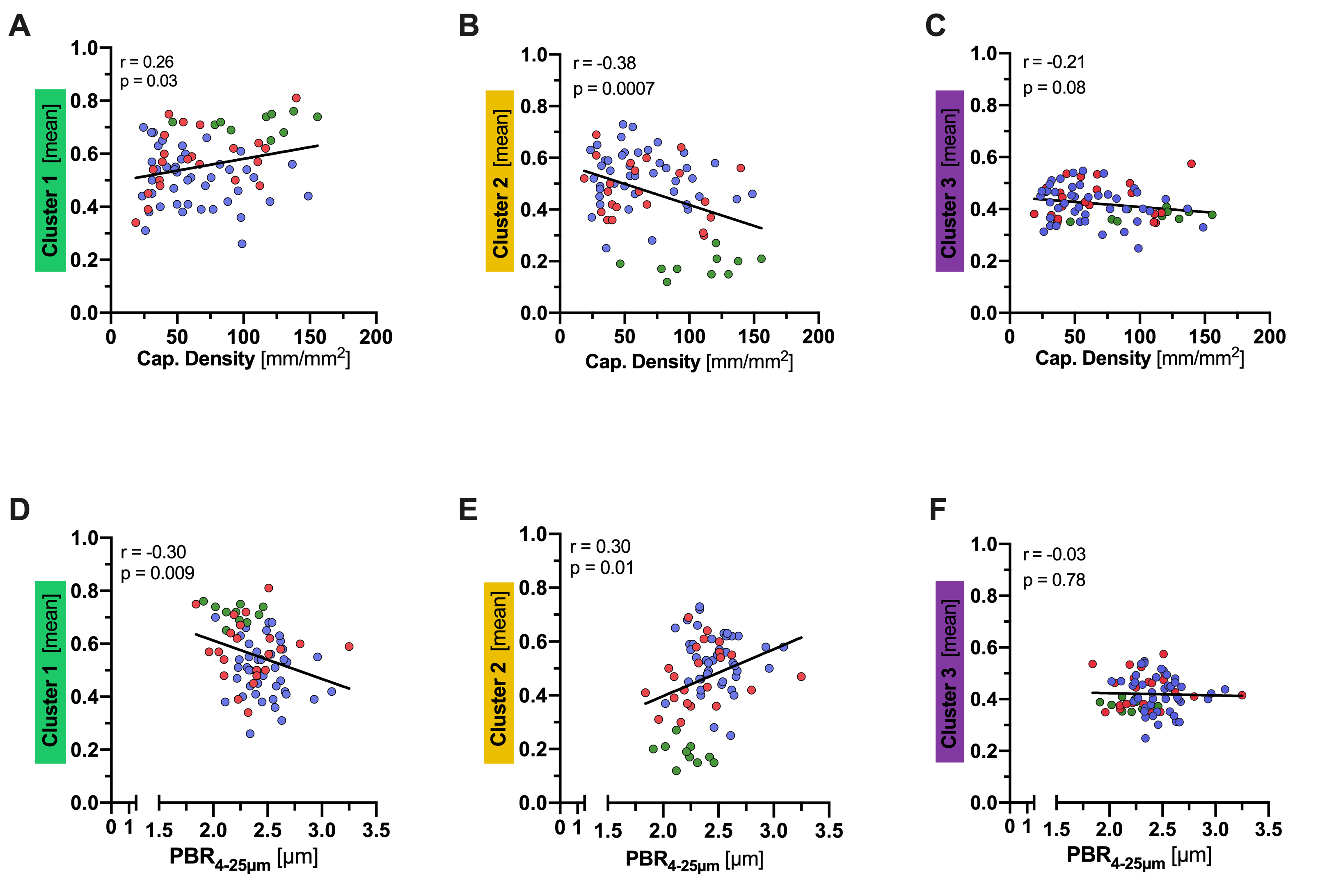

Supplement: Supplementary file 6 — Supplementary file6 (TIFF 857 kb) [file 10456_2022_9843_MOESM6_ESM.tiff]

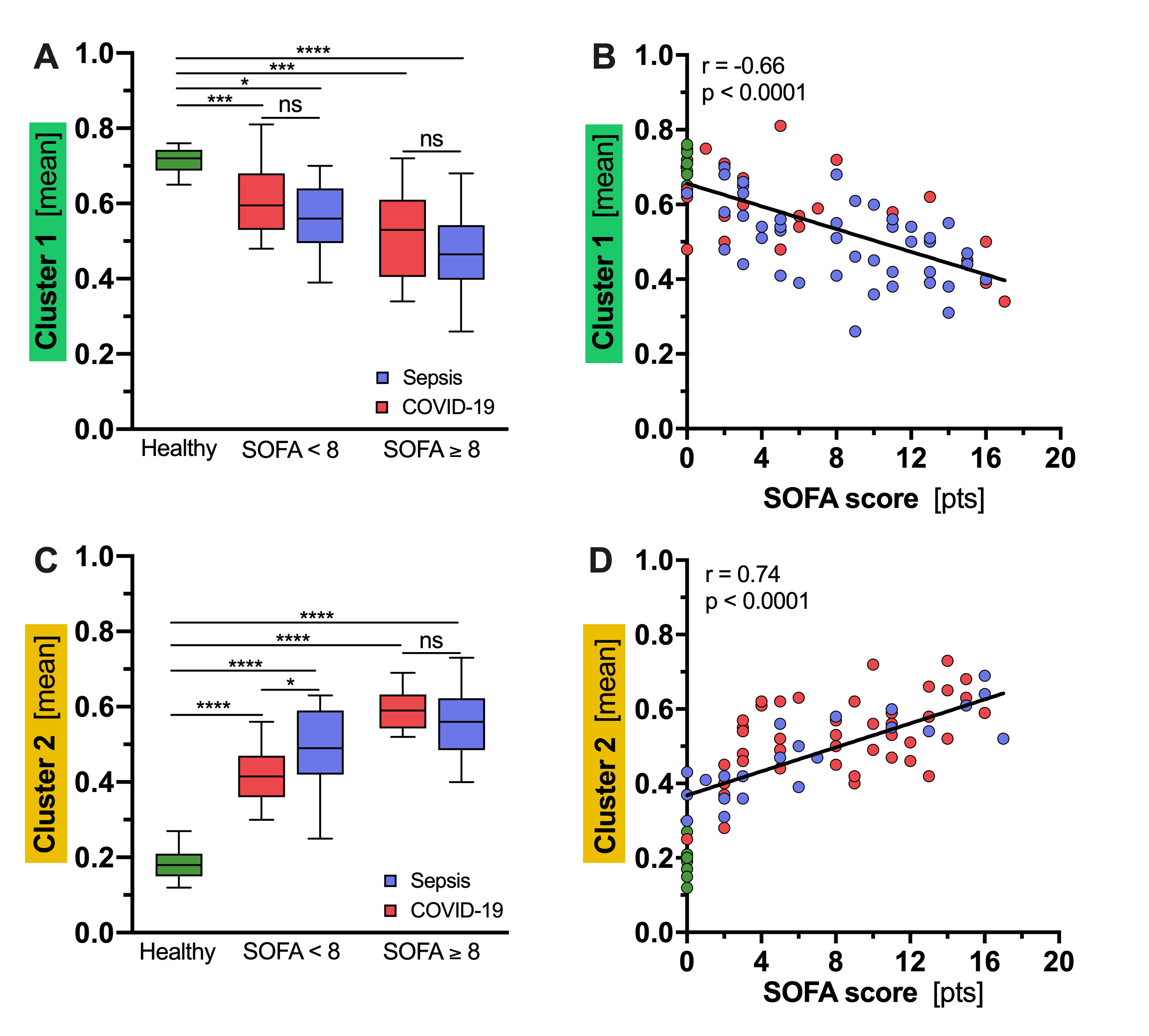

Supplement: Supplementary file 7 — Supplementary file7 (TIFF 586 kb) [file 10456_2022_9843_MOESM7_ESM.tiff]

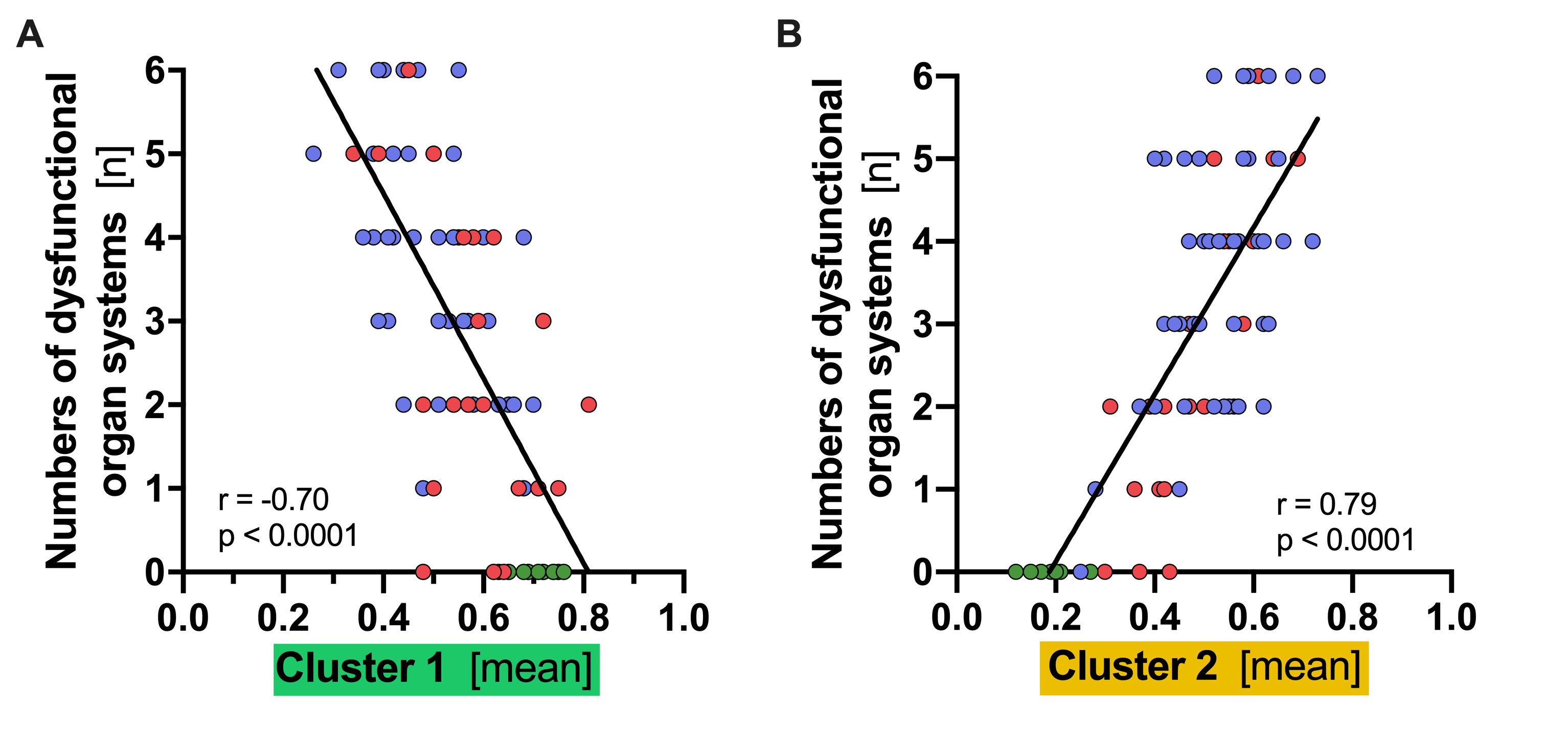

Supplement: Supplementary file 8 — Supplementary file8 (TIFF 439 kb) [file 10456_2022_9843_MOESM8_ESM.tiff]

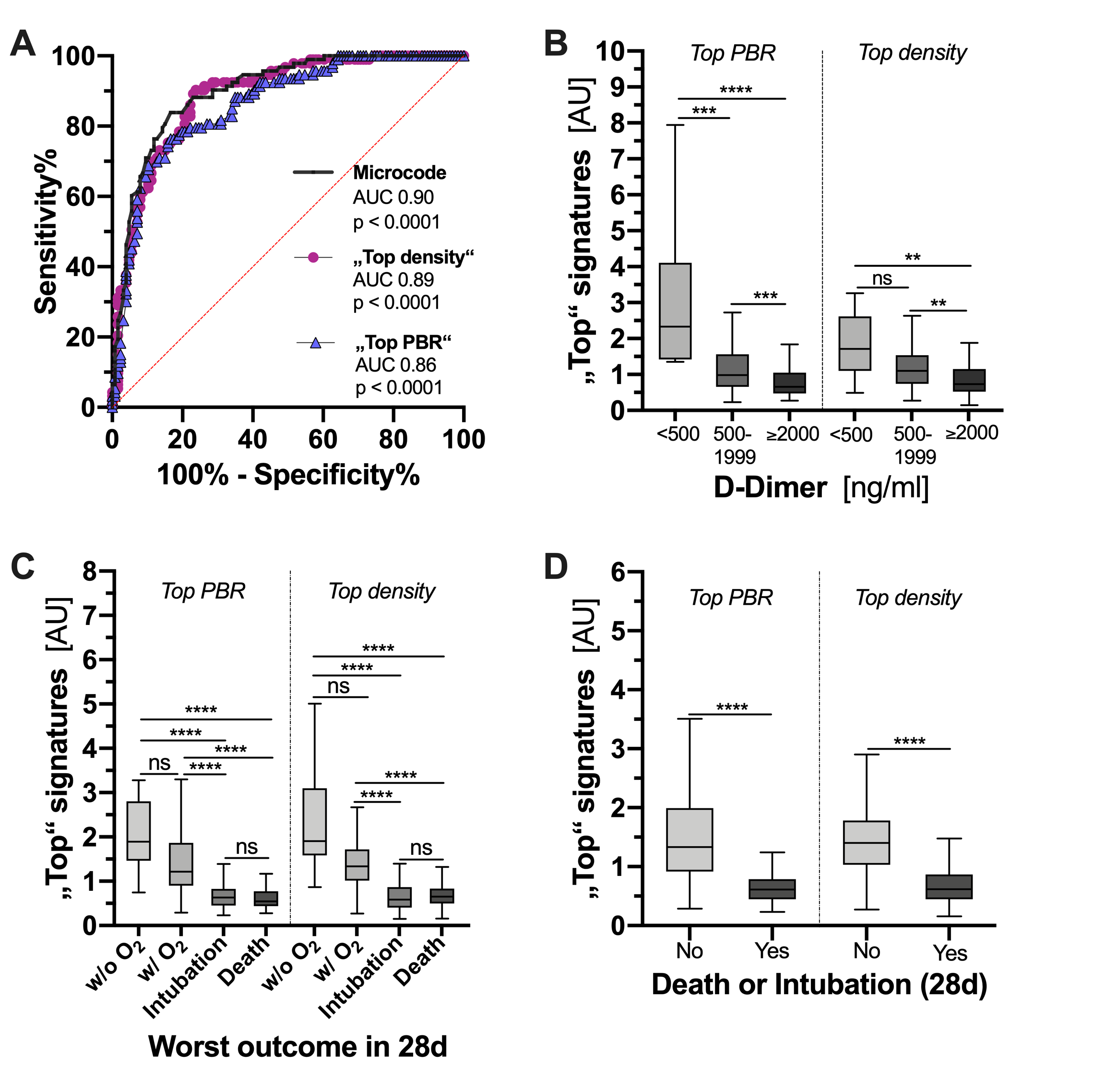

Supplement: Supplementary file 9 — Supplementary file9 (TIFF 586 kb) [file 10456_2022_9843_MOESM9_ESM.tiff]
