# Supplementary material for: Microvascular and proteomic signatures overlap in COVID-19 and bacterial sepsis: the MICROCODE study
Source: Angiogenesis. 2022 Jun 20;25(4):503–15. doi: 10.1007/s10456-022-09843-8 (PMC9208353; doi:10.1007/s10456-022-09843-8)
Supplement: Supplementary file 10 — Supplementary file10 (DOCX 28 kb) [file 10456_2022_9843_MOESM10_ESM.docx]

**Microvascular and proteomic signatures overlap in COVID-19 and bacterial sepsis—the MICROCODE Study**

**Running title:** The MICROCODE Study

Alexandros Rovas^1*^, Konrad Buscher^1*^, Irina Osiaevi^1,2^, Carolin Drost^1^, Jan Sackarnd^3^, Phil-Robin Tepasse^4^, Manfred Fobker^5^, Joachim Kühn^6^, Stephan Braune^7^, Ulrich Göbel^8^, Gerold Thölking^1,9^, Andreas Gröschel^10^, Jan Rossaint^11^, Hans Vink^12^, Alexander Lukasz^1^, Hermann Pavenstädt^1^, Philipp Kümpers^1^

**Supplemental Figures**

**Supplemental Fig. 1:** Optimal number of clusters in training, test and validation set. The elbow method was applied using k means.

**Supplemental Fig. 2:** Principal component (PC) analysis of bacterial sepsis and COVID-19 patients. The ellipses show a probability of 95% that a new datapoint from the same group is located inside the ellipse.

**Supplemental Fig. 3:** Coregulated protein clusters of 184 proteins (red = high positive correlation, blue = high negative correlation) in the test-set. The test cohort was analyzed and visualized as described in Fig. 2. Clustering is based on a dendrogram as described in Fig. 2.

**Supplemental Fig. 4:** Coregulated protein clusters in the external COVID-19 validation cohort from Massachusetts General Hospital (MGH). A public dataset of 219 COVID-19 patients (day 3, 215 positive, 4 negative) was used with 156 serum proteins that were measured using the Olink platform. (A) The validation cohort was analyzed and visualized as described in Fig. 2. (B) Venn diagram demonstrating substantial overlap between cluster 1 (COVID-19/bacterial sepsis dataset) and cluster B (MGH dataset), and between cluster 2 (COVID-19/bacterial sepsis dataset) and cluster A (MGH dataset).

**Supplemental Fig. 5:** Scatter plot and linear correlation between proteomic clusters 1 and 2 in the study population.

**Supplemental Fig. 6: Correlations between proteomic clusters and microvascular parameters.**

The proteins in each cluster were normalized and their means were calculated per subject.

(A, D) Scatter plot of individual cluster 1 means plotted against (A) capillary density and (D) PBR.

(B, E) Scatter plot of individual cluster 2 means plotted against (B) capillary density and (E) PBR.

(C, F) Scatter plot of individual cluster 3 means plotted against (C) capillary density and (F) PBR.

**Supplemental Fig. 7: Associations of cluster 1 and cluster 2 with disease severity and disease entity.** The proteins in each cluster were normalized and their means were calculated per subject. Boxplots showing associations of clusters (A) 1 and (C) 2 with sequential organ failure assessment (SOFA) score after dichotomizing the patient groups based on the median. Scatter plot and linear correlations of clusters (B) 1 and (D) 2 with SOFA score.

ns, not significant

**p* < 0.05, ***p* < 0.01, ****p* < 0.001, *****p* < 0.0001

**Supplemental Fig. 8:** Scatter plots and linear correlations between proteomic clusters 1 (A) and 2 (B) and the number of dysfunctional organ systems in healthy controls (green), bacterial sepsis patients (blue), and COVID-19 patients (red).

**Supplemental Fig. 9:** “Top PBR” and “top density” iterations associated with clinical outcome in the external COVID-19 cohort from Massachusetts General Hospital (*n* = 219). Briefly, the proteins showing the best positive and negative correlations with PBR or capillary density in the present study cohort were tested with respect to the composite endpoint of 28-day mortality and/or intubation in the external COVID-19 cohort. To export a proteomic signature for the microvasculature, the normalized values of the 4 top PBR or top density proteins were added. (A) Receiver operating characteristic curves showing the predictive capacity of the “top PBR” and “top density” protein combinations compared to the *Microcode* signature. (B–D) Boxplots of the “top PBR” and “top density” signature values in relation to (B) corresponding D-dimer values, (C) the worst outcome in the 28 days of hospitalization, and (D) the composite endpoint of 28‑day mortality and/or intubation.

**Supplemental Tables**

**Supplemental Table 1:** Focus of infection and causative pathogens.

| **Focus of infection** (n; %) |  |
| --- | --- |
| Respiratory tract | 21 (48.8) |
| Urinary tract | 6 (14) |
| Prosthesis | 5 (11.6) |
| Gastrointestinal tract | 3 (7) |
| Skin | 3 (7) |
| CNS | 2 (4.6) |
| Heart | 1 (2.3) |
| Unknown | 2 (4.7) |
| **Pathogens (genus)** (n) |  |
| Klebsiella | 10 |
| Staphylococcus | 8 |
| E. Coli | 8 |
| Enterococcus | 6 |
| Streptococcus | 4 |
| Pseudomonas | 2 |
| Others | 13 |

**Supplemental Table 2**: Healthy controls, training, and test cohorts

| **Variables** | **Healthy Controls** | **Training Cohort** | **Test Cohort** | **P value *** |
| --- | --- | --- | --- | --- |
| Number of participants (n) | 10 | 33 | 32 | - |
| Female sex (n; %) | 7 (70) | 10 (30.3) | 7 (21.9) | 0.57 |
| Age (years, median (IQR)) | 51 (27 – 69) | 63 (52 – 77) | 67 (56 – 77) | 0.57 |
| BMI (kg/m^2^, median (IQR)) | 23 (21.5 – 25.8) | 25.5 (23.2 – 29.4) | 25.8 (21.7 - 28) | 0.65 |
| COVID-19 (n; %) | - | 11 (33.3) | 11 (34.4) | 0.99 |
| ICU (n;%) | - | 24 (72.7) | 24 (75) | 0.99 |
| SOFA score(median (IQR)) | - | 8 (3 – 12) | 8 (3 – 13) | 0.71 |
| Number of dysfunctional organs (median (IQR)) | - | 3 (2 – 4) | 3 (2 – 5) | 0.72 |
| Mechanical ventilation (n; %) | - | 19 (57.6) | 13 (40.6) | 0.22 |
| Acute dialysis (n; %) | - | 5 (15.2) | 9 (28.1) | 0.24 |
| Inhospital mortality (n; %) | - | 12 (36.4) | 6 (18.8) | 0.10 |
| **Macrocirculation data** (median (IQR)) | | | | |
| MAP (mmHg) | 92.3 (89.2 – 99.4) | 70 (65 – 85) | 78.7 (73.4 – 89.3) | 0.054 |
| Norepinephrine dose (μg/kg/min) | - | 0.0 (0 – 0.06) | 0.0 (0.0 – 0.08) | 0.86 |
| **Microcirculation data** (median (IQR)) | | | | |
| PBR_4-25µm_ | 2-23 (2.10 – 2.34) | 2.44 (2.30 – 2.54) | 2.4 (2.24 – 2.63) | 0.83 |
| Capillary Density | 118.9 (81.7 – 132.1) | 58.1 (36.2 – 92.6) | 54.0 (38.0 – 88.5) | 0.81 |
| V_RBC_ | 105.2 (93.2 – 1223.0) | 93.7 (81.6 – 102.8) | 95.3 (76.7 – 107.3) | 0.90 |
| MVHS_static_ | 3.4 (2.4 – 4.5) | 1.3 (0.9 – 2.54) | 1.3 (0.98 – 2.15) | 0.99 |
| **Laboratory data** (median (IQR)) | | | | |
| CRP (mg/dl) | 0.5 | 17.9 (11.1 – 23.2) | 19.9 (8.8 – 33.6) | 0.60 |
| Ferritin (µg/l) | 106 (18 – 255) | 712 (349 – 1248) | 630 (245 – 1092) | 0.53 |
| PCT (ng/ml) | 0.05 | 2.4 (0.4 – 12.1) | 3.1 (0.6 – 43.4) | 0.62 |
| Creatinine (mg/dl) | 0.85 (0.68 – 0.95) | 1.2 (0.8 – 2.9) | 1.7 (0.9 – 2.93) | 0.38 |

*p-value was calculated between training and test cohort.

BMI = Body mass index, CRP = C-reactive protein, IQR = interquartile range, MAP = Mean arterial pressure, PCT = Procalcitonin, SOFA score = Sequential Organ Failure Assessment score, PBR = perfused boundary region, V_RBC_ = red blood cell velocity, MVHS = Microvascular Health Score.

**Supplemental Table 3:** A list of unique proteins of Cluster 1, Cluster 2 and Cluster 3. The explanation of the abbreviations can be found online: https://www.olink.com/?s=

| **Cluster 1** | **Cluster 2** | | | |
| --- | --- | --- | --- | --- |
| Flt3L | SLAMF7 | IL18R1 | CX3CL1 | CCL3 |
| GDF2 | PARP1 | IL1ra | MCP3 | HAOX1 |
| FABP2 | ARTN | IL17A | IL27 | ENRAGE |
| CD40L | IL16 | TRAILR2 | HO1 | IL18 |
| TRANCE | IL10 | TGM2 | IL10RA | HBEGF |
| TNFB | MMP10 | LIFR | TNFRSF9 | uPA |
| SOD2 | OPG | TNFSF14 | PRSS8 | FS |
| ADAMTS13 | VEGFA | PAR1 | LOX1 | CCL4 |
| AXIN1 | PGF | OSM | TNFRSF11A | CEACAM8 |
| VEGFD | CSF1 | AGRP | TNF | MMP1 |
| CTRC | CD4 | CD40 | SIRT2 |  |
| CD6 | IL17C | CA5A | MMP12 |  |
| IDUA | CCL20 | IL15RA | CTSL1 |  |
| ANGPT1 | SPON2 | IL6 | CCL19 |  |
| PRSS27 | TNFRSF10A | SORT1 | IL33 |  |
| CXCL5 | REN | FGF23 | Dkk1 |  |
| SCF | CDCP1 | VSIG2 | IL24 |  |
| SRC | FGF5 | IL20 | DCN |  |
| PDGFsubunitB | CCL28 | PAPPA | IL2 |  |
| STK4 | KIM1 | CXCL10 | CCL23 |  |
| TWEAK | IL4RA | 4EBP1 | TM |  |
| TRAIL | PDL1 | TGFalpha | LIF |  |
| DNER | hOSCAR | LAPTGFbeta1 | MCP1 |  |
|  | HGF | TF | TIE2 |  |
|  | TNFRSF13B | SLAMF1 | PIgR |  |
|  | CXCL9 | ACE2 | PRELP |  |
|  | CASP8 | FGF21 | GT |  |
|  | IL8 | Gal9 | BNP |  |
|  | IL10RB | CD5 | PDL2 |  |
|  | XCL1 | PTX3 | ADM |  |
| Cluster 3 | | | | |
| GDNF | THPO | IL20RA | IL22RA1 | SERPINA12 |
| NRTN | IL2RB | CD8A | AMBP | MMP7 |
| CXCL1 | IL13 | CST5 | IL12B | IL1RL2 |
| IFNgamma | MERTK | GH | GLO1 | PSGL1 |
| RAGE | IL4 | MCP2 | DECR1 | FGF19 |
| STAMBP | BetaNGF | THBS2 | ADA | CCL25 |
| CXCL6 | NT3 | HSP27 | IL7 | MARCO |
| TSLP | IgG | IL5 | BMP6 | IL17D |
| IL1alpha | ST1A1 | MCP4 | CCL11 | ITGB1BP2 |
| BOC | CCL17 | NEMO | LEP | FcreceptorIIb |
| CD84 | LPL | CD244 | GIF |  |
